# Supplementary material for: Characterizing Parkinson’s Disease Clinical and Biomarker Interactions in REM Sleep Behavior Disorder
Source: medRxiv. 2025 May 18:2025.05.16.25327469. Preprint. [Version 1] doi: 10.1101/2025.05.16.25327469 (PMC12132163; doi:10.1101/2025.05.16.25327469)
Supplement: Supplementary file 1 [file NIHPP2025.05.16.25327469V1-supplement-1.pdf]

Supplementary Table 1: Comparison of Yale RBD+ Cohort to PPMI, ICEBERG and Oxford Discovery Populations.

	PPMI (USA)				ICEBERG (France)				Oxford (UK)			Yale (USA)			
	Control	iRBD	PDRBD-	PDRBD+	Control	iRBD	PDRBD-	PDRBD+	Control	RBD	PD	Control	RBD	PDRBD-	PDRBD+
N	196	38	145	278	68	50	63	45	296	171	117	15	36	15	18
**Demographic Characteristics**															
M/F	126/70	32/6	82/63	195/83	38/30	45/5	37/26	32/13	145/151	151/20	84/35	13/2	25/11	16/2	11/4
Age (y)	60.7±11.2	69.4±5.5	62.2±9.7	61.2±9.7	62.0±9.1	68.0±5.7	59.6±10.0	65.5±6.4	64.9±10.2	64.7±9.0	66.9±9.1	64.4 ±1.8	68.9±1.1	67.0±1.6	68.5±1.8
Disease duration (y)	-	-	0.6±0.6	0.5±0.5	-	-	1.3±1.0	1.7±1.0					3.4±3.3	1.8 ± 1.1	2.3±2.2
**Autonomic and Non-Motor Aspects of Daily Living**															
MDS-UPDRS I	3.0±3.0	7.1±4.0	4.8±4.0	6.2±4.3	5.0±3.5	9.0±5.0	8.6±3.9	11.1±4.5		9.5±6.5	7.0±4.5	2.5 ± 2.59	7.4± 4.4	5.4 ± 3.9	7.9 ± 4.3
SCOPA-AUT	5.8±3.7	14.9±8.2	7.3±4.8	10.7±6.5	7.2±5.8	12.8±6.3	10.1±6.3	15.7±7.5				5.6 ± 6.5	12.9 ± 6.3	11.7 ± 5.5	13.0 ± 7.0
**Motor and Motor Aspects of Daily Living**															
MDS-UPDRS II	0.5±1.0	2.1±2.4	4.6±3.4	6.3±4.4	1.2±1.7	2.4±2.1	6.8±3.7	9.7±3.3				0.7 ± 1.9	3.6 ± 6.0	4.9± 3.3	8.1 ± 5.0
MDS-UPDRS IIII Off	1.2±2.2	4.7±3.9	19.7±8.1	20.8±9.3	5.6±5.1	11.1±6.0	28.5±7.0	31.2±7.9	1.7±2.7	4.8±6.0	25.7±11.	3.0± 3.4	6.3± 6.7	22.0 9.2	25.8±9.2
**Cognition**															
MoCA	28.2±1.1	25.5±4.2	27.3±2.2	27.1±2.4	27.8±2.0	27.3±2.3	27.5±1.8	27.6±2.3	26.7±2.7	28.1±2.9	25.2±3.3	27.1 ± 1.4	25.9±3.3	27.9±2.2	27.2±2.4
**Sleep**															
RBDSQ	2.8±2.3	9.0±3.0	2.4±1.4	5.0±2.8	-	-		-		-	-	1.1±1.7	10.1±1.7	4.3±2.3	9.6±2.3
**DaT SBR**															
Putamen, contralat	2.1±0.6	-	0.7±0.3	0.7±0.3	-	-		-		-	-	-	1.4±0.5*		
Putamen, ipsilat.	2.1±0.6	-	1.0±0.3	0.9±0.4	-	-		-		-	-	-	1.6±0.6*		
Caudate, contralat.	3.0±0.6	-	1.9±0.5	1.8±0.6	-	-		-		-	-	-	2.6±0.9*		
Caudate, ipsilat.	3.0±0.6)	-	2.2±0.6	2.1±0.6	-	-		-		-	-	-	2.9±0.9*		

Table modified after Di Folco et al[6] Abbreviations: SBR = striatal binding ratio, y = years. Sources: PPMI, ICEBERG: DiFolco et al 2023; Oxford: Barber et al 2017. * Due to lack of motor symptom laterality in most RBD participants, SBR of Yale RBD+ Cohort is reported as mean of right and left for each basal ganglia region. **contra = contralateral (to side of worst symptoms)
